# Polyhexamethylene guanidine phosphate, chloromethylisothiazolinone, and particulate matter are dispensable for stress granule formation in human airway epithelial cells

**DOI:** 10.1080/19768354.2021.1931442

**Published:** 2021-05-26

**Authors:** Arnoldo Cambronero-Urena, Sunkyung Choi, Seri Choi, Kee K. Kim, Eun-Mi Kim

**Affiliations:** a Department of Biochemistry, Chungnam National University, Daejeon, South Korea; b Department of Predictive Toxicology, Korea Institute of Toxicology, Daejeon, South Korea

**Keywords:** Chloromethylisothiazolinone, Particulate matter, Polyhexamethylene guanidine phosphate, Stress granule

## Abstract

Environmental risk factors are recognized as threats to public health. Stress granules (SGs) are non-membranous assemblies of mRNAs and proteins expressed in response to various stressors to promote cell survival. In this study, SG formation was examined to confirm the effects of polyhexamethylene guanidine phosphate (PHMG), chloromethylisothiazolinone (CMIT), and particulate matter (PM10) in airway epithelial cells, A549, HPAEpiC, and BEAS-2B cells. SGs were not observed after CMIT, PHMG, and PM10 treatments, as determined by immunofluorescence microscopy. Moreover, there was no change in the phosphorylation of the translation initiation factor eIF2αfollowing treatment with PHMG, CMIT, and PM10. Taken together, our findings might help determine the biological hazards of these materials.

## Introduction

Globally, air contains different substances from various sources, some of which may be harmful to humans and other living organisms (Kampa and Castanas 2008; Ritz et al. 2019). Among various air pollutants, particulate matter (PM) has been implicated as a source of various health problems, ranging from cardiovascular and pulmonary illnesses to premature mortality (Han 2019). On the contrary, several studies have shown that chemicals such as polyhexamethyleneguanidine (PHMG) and chloromethylisothiazolinone (CMIT), added to household humidifiers as disinfectants, cause fatal lung injury, including interstitial pneumonitis and widespread lung fibrosis(Park et al. 2020). PHMG derivatives are a part of the polymeric guanidine family and are extensively used as antiseptics in various industries (Vitt et al. 2015). Similarly, CMIT has been widely used in biocides, paints, and cosmetics owing to its preservative and disinfectant effects (Go et al. 2020). However, they may induce different cellular responses, such as cellular stress and cytotoxic effects (Kim et al. 2019; Park et al. 2019).

Cells are exposed to various internal and external stimuli when they are a part of a normal tissue or when they are grown in culture, and some of these stimuli induce stress (Poljsak and Milisav 2012). Persistent stress on cells often enhances their susceptibility to pathological conditions, such as cardiovascular abnormalities, chronic neurodegenerative diseases, and cancer (Liguori et al. 2018). After sensing a stress stimulus, the initial response of cells is to defend against and recover from the insult. Cellular responses range from activating pathways that promote survival to inducing programmed cell death that eliminates damaged cells (Fulda et al. 2010).

The formation of stress granules (SGs) is one of the mechanisms involved in re-establishing cellular homeostasis by temporary adaption. SGs are dense aggregates that are formed from messenger RNAs (mRNAs) during translation initiation and are composed of various translation initiation factors, a variety of RNA-binding proteins (RBPs), and several non-RNA-binding proteins (Protter and Parker 2016; Gupta et al. 2017). SGs are assembled under different types of stress, such as hypoxia, nutrient deprivation, and chemical poisoning (Gupta et al. 2017). The assembly usually involves the phosphorylation of serine residue 51 of the alpha subunit of eukaryotic initiation factor 2 (eIF2α), which is a component of the initiation factor eIF2/tRNAi Met/GTP ternary complex. The phosphorylation of eIF2α leads to impaired translational initiation and the consequent polysome disassembly, thereby decreasing protein synthesis (Anderson et al. 2015). Their main function is to protect RNAs from harmful conditions. For example, they minimize energy expenditure, control protein and ribostasis, and improve cell survival under damaging conditions (Mahboubi and Stochaj 2017).

Lung diseases triggered by PHMG, CMIT, and PM10 have been studied from epidemiological and toxicological perspectives. However, SG formation induced by these compounds has not yet been investigated. In this study, we examined the formation of SGs by PHMG, CMIT, and PM10 in airway epithelial cells.

## Material and methods

### Cell culture and chemicals

The human lung adenocarcinoma cell line A549 was cultured in Dulbecco’s modified Eagle medium (DMEM; WELGENE, Gyeongsangbuk-do, Korea) supplemented with 10% fetal bovine serum (FBS; Gibco, NY, USA) and 1% penicillin/streptomycin (Hyclone, Logan, UT, USA). The human pulmonary alveolar epithelial cell line HPAEpiC was cultured using RPM1 1640 medium (WELGENE) supplemented with 10% fetal bovine serum (Gibco) and 1% penicillin/streptomycin (Hyclone). The human pulmonary bronchial epithelial cell line BEAS-2B was cultured with the BEBM^TM^ Bronchial Epithelial Basal Medium (Lonza, Basel, Switzerland). All three cell lines were maintained at 37 °C and 5% CO_2_.

Sodium arsenite (NaAsO_2_) and fine dust (PM10) were purchased from Sigma-Aldrich (St. Louis, MO, USA). Chloromethylisothiazolinone (CMIT) and polyhexamethylene guanidine (PHMG) were purchased from Key Organics (Camelford, UK) and BOC science (Shirley, NY, USA), respectively.

### Cell viability

Cell viability was determined using the Cell Counting Kit (CCK)-8 (Dojindo Laboratories, Kumamoto, Japan). HPAEpiC cells were cultured according to a standard procedure in a 96-well plate and were maintained at 37 °C and 5% CO_2_overnight. After chemical treatment of CMIT and PHMG, CCK-8 solution was added to each well. Cells were then maintained at 37 °C and 5% CO_2_ for 24 h, after which the absorbance readings were obtained at 450 nm using a microplate reader (Molecular Devices EMax Plus, CA, USA).

### Immunofluorescence microscopy

Cells were fixed with 4% paraformaldehyde for 10 min, permeabilized with 2.5% Triton X-100 in phosphate-buffered saline (PBS) for 15 min, blocked with 5% goat serum for 40 min and incubated overnight at 4°C with primary antibodies for G3BP1 (1:500, α-mouse, Santa Cruz Biotechnology, Dallas, TX, USA) and RBFOX2 (1:500, α-rabbit, Bethyl Laboratories, Montgomery, TX, USA). Alexa-488- and Alexa-594-conjugated goat antibodies against mouse and rabbit IgG (1:1000, Thermo Fisher Scientific) were used as secondary antibodies. Nuclei were stained with 4,6-diamidino-2-phenylindole (DAPI, Thermo Fisher Scientific). Images were analyzed with LSM 880 Airyscan confocal laser-scanning microscope (Carl Zeiss, Oberkochen, Germany) and with the software Zen® Blue edition (Zeiss).

### Immunoblot analysis

Whole cell lysates were prepared with M-PER buffer (Thermo Scientific, Waltham, MA, USA) supplemented with a protease-inhibitor cocktail (Roche Applied Science, Schlieren, Switzerland). Protein samples were denatured and reduced by sodium dodecyl sulfate (SDS) and β-mercaptoethanol, respectively. Protein was separated on SDS-polyacrylamide gels and transferred onto a nitrocellulose membrane, which were blocked with 5% nonfat milk and incubated overnight at 4 °C with primary antibodies for GAPDH (1:2000, α-mouse, Santa Cruz Biotechnology) and p-eIF2α (1:1000, α-rabbit,Cell Signaling Technology, Danvers, MA, USA). After washing with PBS, the blots were incubated with peroxidase-conjugated secondary antibodies for 1 h at room temperature. The Super Signal® West Dura Extended Duration Substrate (Thermo Scientific) was used to detect the binding of secondary antibodies. Images were analyzed with WSE-6100 Luminograph I (Atto Corporation, Tokyo, Japan).

## Results and discussion

### Effect of CMIT, PHMG, and PM10 on the viability of airway epithelial cells

**Figure 1.**
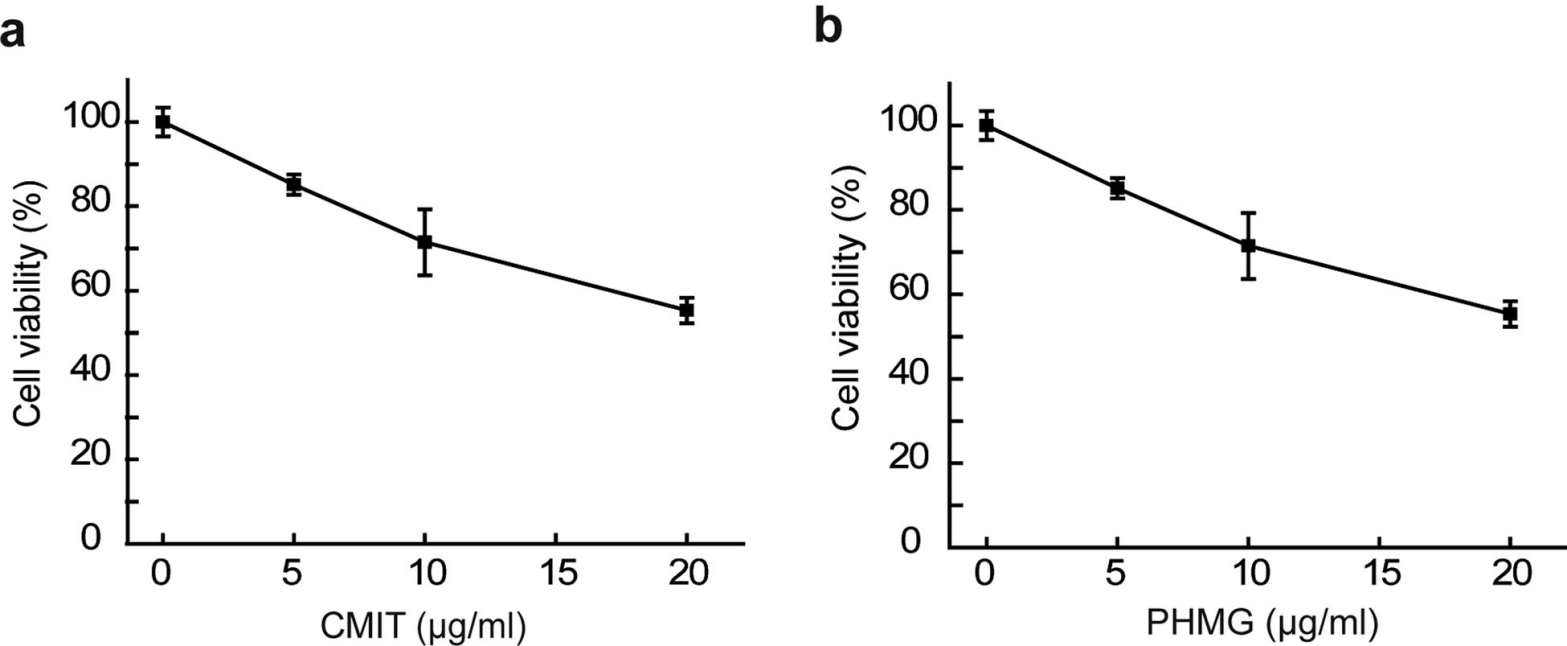
Cytotoxicity profiles of CMIT and PHMG in HPAEpiC cells. HPAEpiC cells were treated with indicated concentrations of CMIT **(a)** and PHMG **(b)** for 24 h, and cell viability was determined by the CCK Assay.

Cellular stress, including chemical compounds, can trigger SG formation by affecting translation, proteasome activity, or other endogenous stressors. However, the action of these compounds may introduce other complicated cellular responses, such as cytotoxic effects, which make it difficult to study their role in SG formation(Hu et al. 2017). In this study, we investigated the effects of environmental risk factors, PM10, and humidifier disinfectants, such as CMIT and PHMG, on SG formation in airway epithelial cells. First, the effects of PM10 treatments on cell viability were evaluated using the CCK assay in HPAEpiC cells. The viability of cells reduced to 73.6% and 55.3% after treatment with CMIT 10 and PHMG 20 μg/mL, respectively (Figure 1). Unexpectedly, no obvious cytotoxicity of PM10 was observed until a concentration of 2 μg/mL (data not shown). CMIT has been reported to induce toxicity to the lungs by inhibiting cell proliferation via p53/p21 related cell cycle arrest and apoptosis associated with upregulation of the MAPK pathway (Lee et al. 2019). Moreover, PHMG has been shown to induce apoptosis via various pathways such as DNA and membrane damage (Park et al. 2018). PM10 has been implicated with oxidative stress induced-cell toxicity by increasing reactive oxygen species (ROS) production (Seok et al. 2018). Taken together, these results help better understand the cytotoxicity profile of CMIT, PHMG, and PM10 in airway epithelial cells.

### CMIT, PHMG, and PM10 treatments did not trigger SG formation

**Figure 2.**
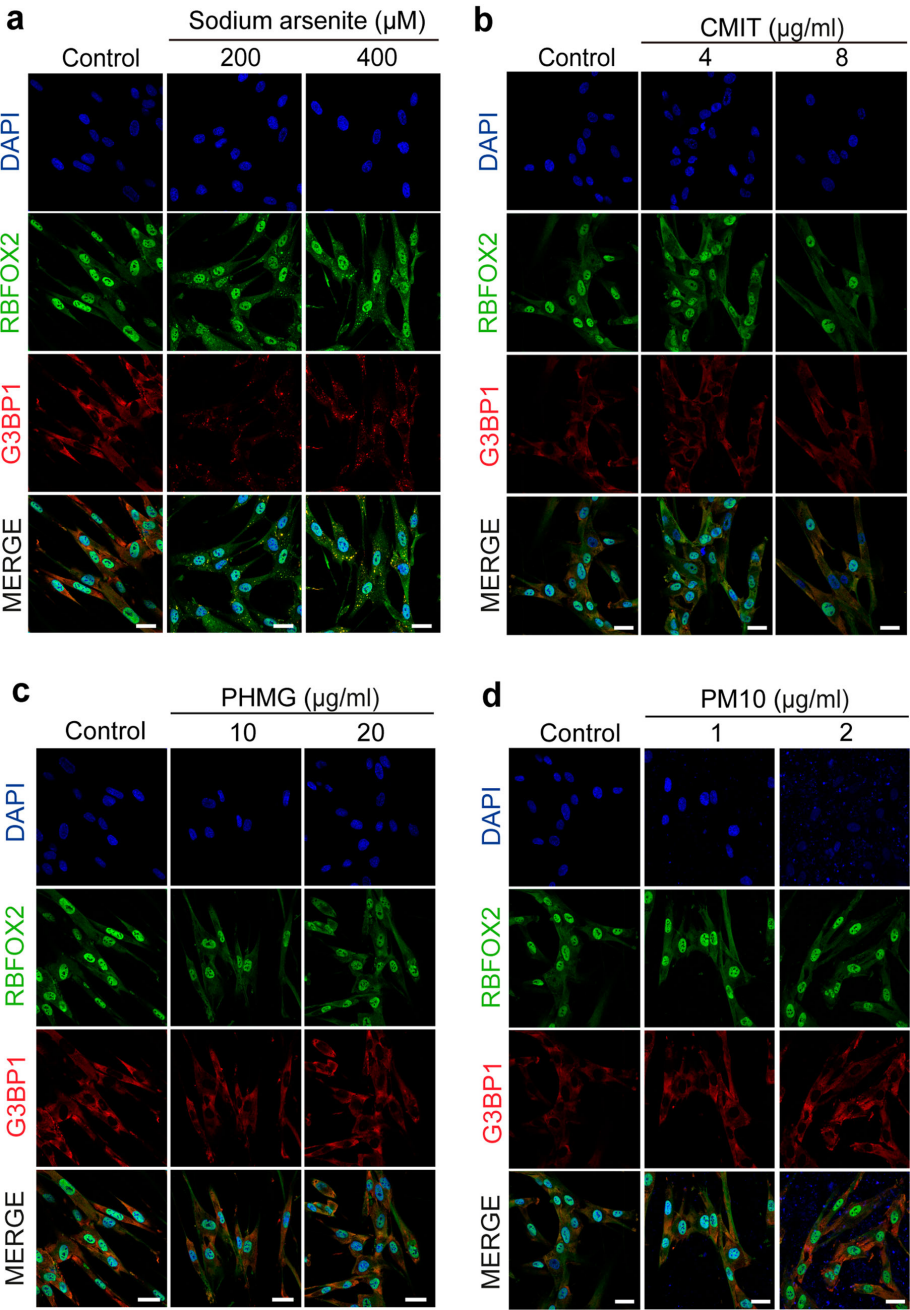
CMIT, PHMG and PM10 do not trigger SG formation. Immunofluorescence images of G3BP1 (red) and RBFOX2 (green) after treatment with200 and 400 μM sodium arsenite **(a)**, 4 and 8 μg/mlCMIT **(b)**, 10 and 20 μg/ml PHMG **(c)**, and 1 and 2 μg/ml PM10 for 1h **(d)**. DAPI (blue) staining represents the nuclei. Scale bar, 20 μm.

SGs contain various RNA-binding proteins and mRNAs and that SGs are able to regulate gene expression, it is necessary to investigate the function of RNA-binding proteins in SGs. We previously reported that RNA-binding motif protein 9 (RBFOX2) was localized in SGs with cell cycle-related mRNAs under several stress conditions (Park et al. 2017; Choi et al. 2019). G3BP1 is a stress granule-resident protein that nucleates stress granule assembly and is well known as a SGs marker (Kedersha et al. 2005). To gain further insights into the assembly of SGs, we examined the subcellular localization of RBFOX2 and G3BP1 by immunofluorescence microscopy after CMIT, PHMG, and PM10 treatments. Sodium arsenite is commonly used as an oxidative stress agent because it has been shown to induce SG formation in different cell types (Arimoto-Matsuzaki et al. 2016). We used sodium arsenite as a positive control to induce SGs formation. Here, sodium arsenite induced the formation of cytoplasmic RBFOX2 granules in HPAEpiC, A549, and BEAS-2B cells, as confirmed by the co-localization of RBFOX2 with the SG marker G3BP1 (Figure 2, Figure S1 and Figure S2). These results suggest that HPAEpiC, A549, and BEAS-2B cells form SGs when exposed to external stimuli.

Next, we examined whether CMIT, PHMG, and PM10 treatments induced the formation of RBFOX2-containing SGs in airway epithelial cells. SG formation was not detected in HPAEpiC, A549, and BEAS-2B cells after CMIT, PHMG, or PM10 treatment (Figure 2, Figure S1 and Figure S2), suggesting that CMIT, PHMG, or PM10 could not induce SG formation in A549, HPAEpiC, and BEAS-2B cells. Exposure to CMIT induces cellular responses, such as apoptosis, inflammation, and oxidative stress (Lee et al. 2019). Furthermore, PHMG has been reported to induce endoplasmic reticulum (ER) stress (Kim et al. 2019). PM10 has been associated with inflammation, ER stress, and oxidative stress (Aztatzi-Aguilar et al. 2018; Familari et al. 2019). Both ER and oxidative stresses have been shown to induce SG formation (Arimoto-Matsuzaki et al. 2016; Mahboubi and Stochaj 2017). Thus, SG formation might be triggered by the chemicals via these pathways, but SGs were not induced. These chemicals might trigger other biochemical pathways, instead of the SG formation pathway.

**Figure 3.**
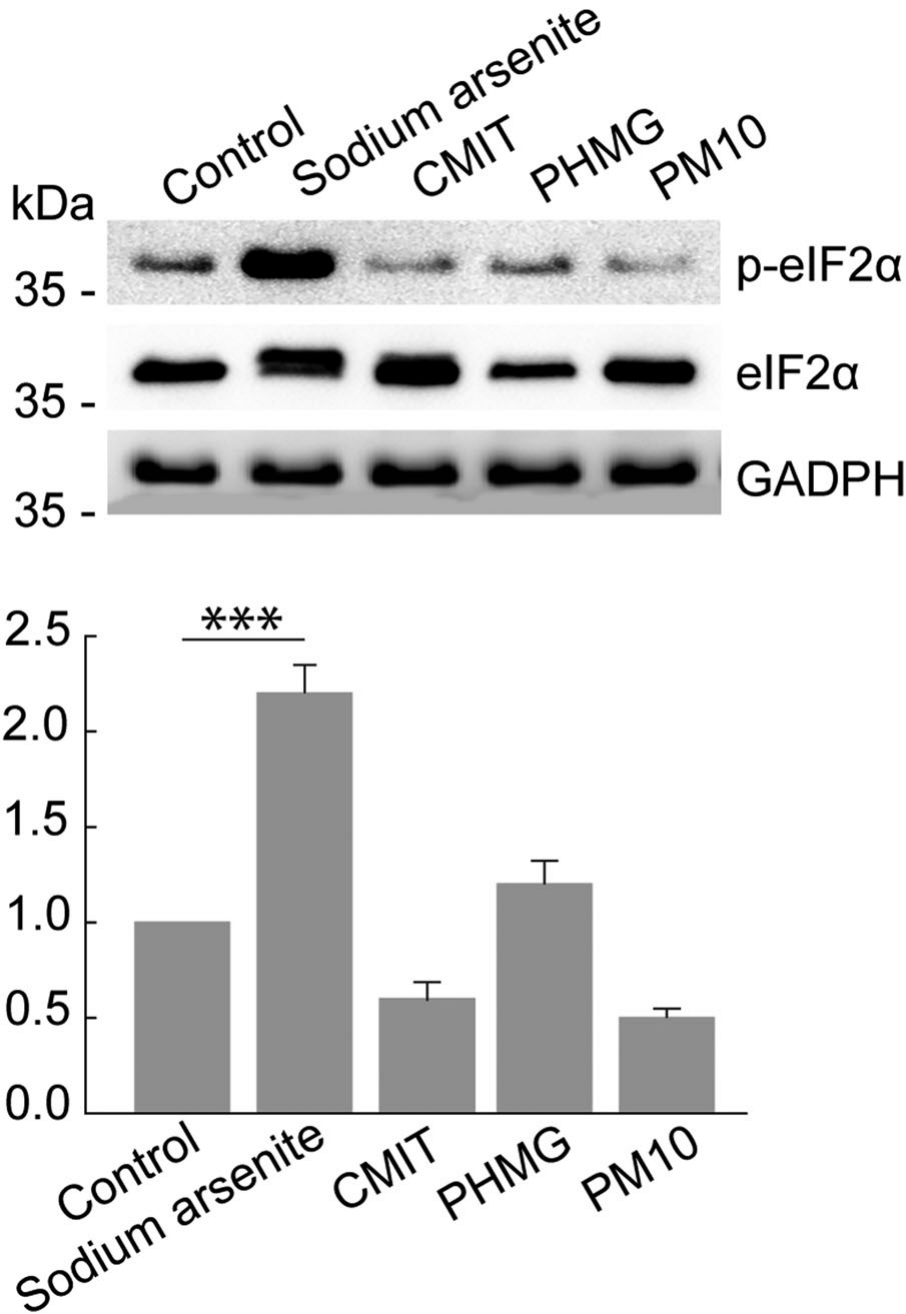
Phosphorylation of eIF2α is affected under cellular stresses in HPAEpiC cells. Cells were treated with 400 μM sodium arsenite, 8 μg/ml CMIT, 20 μg/ml PHMG and 2 μg/ml PM10 for 1h. Cell lysates were analyzed by immunoblot analysis using anti-phospho-eIF2αand anti-eIF2αantibodies. The values under the gels denote the relative intensities of the phospho-eIF2α/eIF2αprotein bands. GADPH served as a loading control.*p < 0.05 compared with the corresponding control.

Besides the type of stress that triggers SG formation, the assembly typically involves the phosphorylation of eIF2α at serine 51. The phosphorylation of eIF2α leads to the inhibition of translation initiation in different ways, resulting in SG formation (Hu et al. 2010). To elucidate SG assembly, the immunoblot analysis was performed to examine eIF2α phosphorylation. As previously reported in various cells (Brown et al. 2011; Park et al. 2017) sodium arsenite treatment induced eIF2α phosphorylation in A549, HPAEpiC, and BEAS-2B cells (Figure 3 and Figure S3). The band intensity for eIF2α phosphorylation did not seem to increase after treatment with CMIT or PM10, compared with the control. These results are in line with the immunofluorescence microscopy observations (Figure 2, Figure S1 and Figure S2). Interestingly, PHMG treatment clearly reduced eIF2α phosphorylation in HPAEpiC and BEAS-2B cells (Figure 3 and Figure S3b). Taken together, these findings suggest that cellular stress induced by CMIT, PHMG, and PM10 does not trigger stress granule assembly in airway epithelial cells.

It is important to note that although SGs were not observed after exposure to CMIT, PHMG, or PM10, these chemicals may trigger SG assembly under other conditions or combined with other stressors, such as a viral infection. These scenarios should be assessed in further research. Under such circumstances, SG formation might play a fundamental role in the cellular stress response. SG formation can affect biological responses in two main ways. First, it has been proposed that recruitment of specific mRNAs might promote their interaction with translation factors to enhance assembly of translation initiation complexes. In contrast, sequestration of particular cellular components could limit their interactions with bulk cytosol (Buchan and Parker 2009; Protter and Parker 2016).

SG formation was not apparently induced by exposure to CMIT, PHMG, or PM10 according to immunofluorescence microscopy and immunoblot analysis for phospho-eIF2α protein levels. However, we did confirm that p-eIF2α expression decreased upon PHMG treatment in HPAEpiC and BEAS-2B cells. Phosphorylation of eIF2α acts as a molecular switch that determines cell fate in response to oxidative stress. Genetic loss of the upstream eIF2α kinases PERK and GCN2, or genetic impairment of p-eIF2α increases susceptibility to cell death by oxidative stress (Rajesh et al. 2015). The exact meaning of reduced p-eIF2α expression in airway epithelial cells exposed to PHMG should be evaluated in further research. To date, studies on lung diseases caused by CMIT, PHMG, and PM10 have focused on toxicological and epidemiological questions. The present study focused on involvement of these chemicals in SG formation, and the results offer further insights into the biological effects these materials might have that could impact human health.
